# MicroRNA-193a-5p Rescues Ischemic Cerebral Injury by Restoring N2-Like Neutrophil Subsets

**DOI:** 10.1007/s12975-022-01071-y

**Published:** 2022-07-29

**Authors:** Ziping Han, Lingzhi Li, Haiping Zhao, Rongliang Wang, Feng Yan, Zhen Tao, Junfen Fan, Yangmin Zheng, Fangfang Zhao, Yuyou Huang, Yue Tian, Guangwen Li, Yumin Luo

**Affiliations:** 1 Institute of Cerebrovascular Diseases Research and Department of Neurology, Xuanwu Hospital of Capital Medical University, 45 Changchun Street, Beijing, 100053 China; 2 Beijing Key Laboratory of Translational Medicine for Cerebrovascular Diseases, Beijing, China; 3 Department of Neurology, The Affiliated Hospital of Qingdao University, No.16 Jiangsu Road, Qingdao, China; 4 Beijing Institute for Brain Disorders, Beijing, China

**Keywords:** miR-193a-5p, Cerebral ischemic injury, N2-like neutrophil, UBE2V2

## Abstract

Circulating neutrophils are activated shortly after stroke and in turn affect the fate of ischemic brain tissue, and microRNAs (miRNA) participate in regulating neuroinflammation. We probed the role of neutrophilic miRNA in ischemic stroke. miR-193a-5p was decreased in circulating neutrophils of acute ischemic stroke (AIS) patients and healthy controls. In another set of AIS patients treated with recombinant tissue plasminogen activator, higher neutrophilic miR-193a-5p levels were associated with favorable outcomes at 3 months and non-symptomatic intracerebral hemorrhage. An experimental stroke model and human neutrophil-like HL-60 cells were further transfected with agomiR-193a-5p/antagomiR-193a-5p or ubiquitin-conjugating enzyme V2 (UBE2V2)-siRNA prior to model induction for in vivo and in vitro studies. Results of 2,3,5-triphenyl tetrazolium chloride staining and neurological function evaluations at post-experimental stroke showed that intravenous agomiR-193a-5p transfusion protected against ischemic cerebral injury in the acute stage and promoted neurological recovery in the subacute stage. This protective role was suggested to correlate with neutrophil N2 transformation based on the N2-like neutrophil proportions in the bone marrow, peripheral blood, and spleen of the experimental stroke model and the measurement of neutrophil phenotype-associated molecule levels. Mechanistically, analyses indicated that UBE2V2 might be a target of miR-193a-5p. Cerebral injury and neuroinflammation aggravated by miR-193a-5p inhibition were reversed by UBE2V2 silencing. In conclusion, miR-193a-5p protects against cerebral ischemic injury by restoring neutrophil N2 phenotype-associated neuroinflammation suppression, likely, in part, via UBE2V2 induction.

**Supplementary Information:**

The online version contains supplementary material available at 10.1007/s12975-022-01071-y.

## Introduction

Shortly after ischemic stroke onset, immune cells infiltrate the ischemic brain, and this robust inflammatory response exacerbates ischemic injury [1–3]. Clinical trials aimed at indiscriminately blocking neutrophil access to the brain parenchyma post-stroke have failed to improve outcomes for ischemic stroke (IS) patients [4–6], in accordance with the previously ignored heterogeneity of neutrophils post-stroke [7, 8]. Recently, increasing evidence has highlighted the alternative phenotypes and functions of circulating neutrophils in patients with IS [9] and that activating/deactivating the mechanisms responsible for neutrophil phenotypic transformation has neuroprotective effects post-stroke. Moro’s team [10] first reported that reprogramming neutrophils toward an N2 phenotype by activating peroxisome proliferator-activated receptor-γ nuclear receptor (PPARγ) alleviates neuroinflammation and increases neutrophil clearance using a permanent middle cerebral artery occlusion model. Toll-like receptor 4 (TLR4) blockage also transforms neutrophils to an N2 phenotype after stroke, mitigating cerebral ischemic injury [11]. Moreover, preclinical evidence using mouse models of transient cerebral ischemia shows that all-trans-retinoic acid prophylactic application can promote neutrophil polarization to an N2 phenotype and inhibit neutrophil accumulation in brain lesions by targeting signal transducer and activator of transcription 1 (STAT1), lessening cerebral ischemic injury [12]. Hence, modulating neutrophil phenotypic transformation post-stroke is a promising strategy for alleviating inflammatory responses, enhancing neuroprotective effects, and promoting functional recovery after cerebral ischemic injury.

MicroRNAs (miRNAs) are small noncoding RNAs composed of 20 ~ 25 nucleotides, which can target multiple mRNAs simultaneously and regulate approximately 30–90% of human gene expression by promoting mRNA degradation or inhibiting translation [13]. Evidence supports the critical role of miRNAs in cerebral ischemia pathophysiology [14, 15]. We and other teams have shown that miRNAs are implicated in neuroinflammation post-IS. Specifically, miR-155 inhibition significantly alters the expression of inflammation-associated cytokines and molecules post-experimental stroke, affecting inflammation after cerebral ischemia [16]. Furthermore, antagomiR‐494 application alters Th1‐mediated neurotoxicity post-stroke by regulating the HDAC2–STAT4 cascade [17] and inhibits neutrophil infiltration and their shift to an N1 phenotype after cerebral ischemic injury by decreasing the expression of multiple MMPs [17]. Moreover, miR-15a/16–1 inhibition alleviates ischemic brain injury by suppressing IL-6, MCP-1, VCAM-1, and TNF-α [18], and miR-1906 application can ameliorate ischemic injury in experimental stroke by directly modulating the inflammatory initiator TLR4 [19]. Collectively, we assumed that circulating neutrophilic miRNAs might participate in neutrophil N2-skewed neuroprotection post-stroke, and we probed the underlying mechanism.

## Methods

### Human Samples

The collection of human samples complied with the Declaration of Helsinki and was approved by the Research Ethics Committee Review Board of Xuanwu Hospital, Capital Medical University, Beijing, China (registration no. ChiCTR-NCT-03577093). Written or informed consent was obtained from all enrolled individuals for the study of blood samples. The acute ischemic stroke (AIS) patient cohort was recruited at Xuanwu Hospital from November 2018 to November 2019. Inclusion criteria for AIS patients were as follows: (1) admitted within 6 h from symptom onset, (2) verified diagnosis of IS via brain magnetic resonance imaging or repeated computed tomography. Patients with acute myocardial infarction, severe heart failure, malignant tumors, renal disease, immune diseases, or other neurological diseases were excluded. Matched healthy volunteers were recruited from the Medical Examination Center of Xuanwu Hospital. Upon admission, patients underwent standard neurological and general medical assessments, and IS was diagnosed following standard guidelines. Incidents of symptomatic intracranial hemorrhage (sICH) were recorded among patients receiving recombinant tissue plasminogen activator (rtPA) treatment. All patients received follow-up care, and the modified Rankin Scale (mRS) score at 3 months was adopted to evaluate their functional prognosis. Three patients diagnosed with AIS and three matched healthy volunteers were selected for the microRNA array project. A subset of 43 AIS patients and 27 matched healthy volunteers were included for validation. Another subset of 128 AIS patients receiving rtPA treatment was included to evaluate its clinical significance.

### miRNA Sequencing, RT-qPCR Validation, and Bioinformatics Analyses

Total RNA from circulating neutrophils obtained from AIS patients and healthy volunteers was isolated for the miRNA sequencing library and subsequent qPCR verification. Briefly, after 3′- and 5′-adaptor ligation, cDNA synthesis, and PCR amplification, 15–35 nt small RNAs were selected and purified for library preparation. Libraries were denatured as single-stranded DNA, captured by Illumina flow cells, amplified as clusters in situ, and finally sequenced using the Illumina NextSeq platform, according to the manufacturer’s instructions. miRNA with a |fold-change|> 1.5 and *p* < 0.05 was considered differentially expressed.

Subsequent RT-qPCR analyses were adopted to validate expression levels of some miRNAs from the miRNA sequencing analysis. The prediction of miRNA-enriched pathways was performed based on miRBase. Database for Annotation, Visualization, and Integrated Discovery (DAVID) functional annotation and the KEGG database were adopted to obtain target gene-enriched pathways. *p* < 0.05 was the criterion for statistical significance.

### Animals

C57BL/6 J mice (20 ± 2-g body weight) were purchased from Vital River Laboratory Animal Technology Co., Ltd. (Beijing, China). They were raised under standard environmental conditions of 50–60% humidity, 22–24 °C, and a 12 h:12 h light:dark cycle in Xuanwu Hospital, with standard laboratory feeding accessible throughout the entire experiment. All animal studies were approved by the Institutional Animal Care and Use Committee of Capital Medical University and performed according to the international and national law and policies (ARRIVE guidelines and the Basel Declaration including the 3Rs concept) [20]. Animal allocation was random and experimenters were blinded to this.

### Transient Middle Cerebral Artery Occlusion (MCAO) Surgery and Groups

The MCAO surgery (experimental stroke model) was conducted through intraluminal occlusion of the right middle cerebral artery, as reported previously [21]. Briefly, the mouse body temperature was maintained at 37.0 ± 0.5 °C with a heat lamp during the entire surgical procedure; mice were anesthetized with isoflurane, and a silicone nylon filament with a 0.19-mm diameter silicon tip (Cat#: 701934PK5Re; Doccol Corporation, Sharon, MA, USA) was inserted into the right middle cerebral artery for 45 min to block cerebral blood flow; the suture was then removed to allow reperfusion (to simulate ischemia/reperfusion injury in vivo). To confirm occlusion of the middle cerebral artery, a transcranial laser Doppler (LDF, PeriFlux System 5000; Perimed, Sweden) was adopted to monitor local cerebral blood flow in the MCAO model. Sham-operated mice were subjected to a similar surgery without suture insertion. All attempts were made to minimize the number and suffering of animals in this study.

Animals were randomly allocated into five groups as follows: (1) sham group + control, mice injected with negative control miRNA subjected to sham MCAO surgery; (2) MCAO + control, mice injected with negative control miRNA subjected to MCAO surgery; (3) MCAO + agomiR-193a-5p, mice injected with mus-agomiR-193a-5p subjected to MCAO surgery; (4) MCAO + antagomiR-193a-5p, mice injected with mus-antagomiR-193a-5p subjected to MCAO surgery; (5) MCAO + antagomiR-193a-5p + UBE2V2-siRNA, mice injected with mus-antagomiR-193a-5p and mus-UBE2V2-siRNA subjected to MCAO surgery. Mus-agomiR-193a-5p, mus-antagomiR-193a-5p, mus-UBE2V2-siRNA, and negative control miRNA (GenePharma, Suzhou, China; listed in Table S1) were intravenously administered to the mice via the tail vein before MCAO/sham surgery, and the needle of the injection syringe was held in situ for several minutes before it was slowly withdrawn.

### Measurement of Brain Infarct Volume

The mouse cerebral infarct volume was evaluated at 24 h and 7 days after MCAO operation. Mice were first anesthetized with 1% pentobarbital sodium and perfused with 0.9% saline. They were then decapitated, and their brains were collected and coronally sliced into 61-mm slices in a brain matrix on ice. The brain slices were subsequently incubated with 2% 2,3,5-triphenyl tetrazolium chloride (TTC; T8877; Sigma-Aldrich, USA) at 37 °C for 10 min and then fixed in 4% paraformaldehyde to determine the infarction volume. The brain pictures were then analyzed with ImageJ software. To correct for brain swelling, the relative infarct territory was determined by subtracting the area of non-infarcted tissue in the ipsilateral hemisphere from that in the contralateral hemisphere. The infarct volume was calculated by integrating the infarct areas of all six slices of each brain [22].

### Human HL-60 Cells and Transfection

Human neutrophil-like HL-60 cells (ATCC, Rockville, MD, USA) were cultured in complete medium (RPMI-1640 medium containing 10% FBS and 1% penicillin–streptomycin) in a humidified atmosphere of 5% CO_2_/95% air at 37 °C. Cells were used for experiments after fewer than 10 passages. HL-60 cells were homogeneously divided into five groups (5 × 10^5^/ml) in 12-well plates as follows: (1) control miRNA (20 μM), (2) LPS (1 ng/ml) + control miRNA (20 μM), (3) LPS (1 ng/ml) + agomiR-193a-5p (20 μM), (4) LPS (1 ng/ml) + antagomiR-193a-5p (20 μM), (5) LPS (1 ng/ml) + antagomiR-193a-5p (20 μM) + UBE2V2 siRNA (20 μM); corresponding interventions were then applied. First, HL60 cells were transfected with hsa-agomiR-193a-5p, hsa-antagomiR-193a-5p, hsa-UBE2V2 siRNA, and negative control miRNA (GenePharma, Suzhou, China; listed in Table S1) and cultivated for 48 h. Then, HL-60 cells were stimulated with LPS for 6 h in serum-free medium, and then, complete medium was reapplied and cells were cultured for 24 h. Finally, 500 μl of supernatant was collected for further ELISA experiments, and the remaining cells were collected for RT-qPCR.

### RNA Extraction and RT-qPCR

Total RNA was extracted from cell and brain tissue samples using TRIzol Reagent (Cat#15,596,026; Invitrogen, USA) according to the manufacturer’s instructions. After spectrophotometric quantification with a nanodrop, 1 μg of total RNA in a final 20 μl volume was used for reverse transcription with a HiScript III 1st Strand cDNA Synthesis Kit (Vazyme, Nanjing, China) and miRNA First-Strand cDNA Synthesis (tailing Reaction; Cat#B532451; Sangon Biotech, Shanghai, China), according to the manufacturers’ instructions. Total cDNA was used for RT-qPCR with the Taq Pro universal SYBR qPCR Master Mix (Vazyme, Nanjing, China) in a StepOne Plus Real-time PCR System (Roche, LightCycler 480 II). The expression of universal U6 was used as a control for miR-193a-5p, and human *GAPDH* or mouse *β-actin* was used as a control for the target mRNA to calibrate the original concentration of cell or tissue mRNA. The 2 − ΔΔCT method was used for target gene expression relative quantification. Each quantitative PCR assay was performed in triplicate and independently repeated three times. Primer sequences are listed in Table S1.

### Flow Cytometry

For flow cytometry, blood cell lysis buffer (eBioscience, San Diego, CA, USA) was added to bone marrow cells, peripheral blood, and spleen cells of different groups of mice and incubated for 15 min at 4 °C. Then, cells were centrifuged and suspended in buffer containing 2% FBS in PBS and stained using the following fluorescently labeled antibodies purchased from BD Bioscience for 30 min: Ly6G-PE, CD11b-FITC, CD206-APC, and isotype controls (FITC, PE, and APC). After filtering through a 40-μm nylon cell strainer, samples were detected using an LSRFortessa SORP flow cytometer (BD, NJ, USA). Ly6G^+^CD11b^+^ cells were gated as neutrophils, and data analysis was implemented with FlowJo software (Ashland, OR, USA).

### Brain Tissue, Peripheral Blood, and Cell Supernatant ELISA

Brain tissues from different groups of mice were lysed in PBS to obtain homogenates; peripheral blood was drawn into heparin-anticoagulant tubes and then centrifuged to obtain the supernatant, and the supernatant of HL-60 cells subjected to different treatments was also collected. According to the manufacturer’s instructions, TGF-β, TNF-α, IL-10, and IL-1β ELISA kits (Neobioscience, Shenzhen, China) were adopted to detect respective molecule levels in different groups of brain tissue, peripheral blood, and HL-60 cell supernatant samples.

### Neurological Function Scoring

Neurological function was assessed by the beam walking test, adhesive removal test, and mouse neurological severity scores (mNSSs). Pre-operative training was performed for three consecutive days, and post-operative testing was performed at 0, 1, 3, 5, and 7 days after cerebral ischemic injury. For the beam walking test, mice were placed on the beam (0.8 cm × 120 cm × 30 cm) and given sufficient time (approximately 5 min) to complete the task; then, the times at which their hind legs fell off within 1 m were recorded [23]. The mNSS test is composed of motor, sensory, balance, and reflex evaluation. A higher neurological deficit score indicates more severe injuries [24]. In the adhesive removal test, a small black adhesive tape strip (30 × 40 mm) was stuck to the mouse paw, and the time required to remove it was recorded three times [25].

### Immunofluorescence (IF) and Fluorescence in Situ Hybridization (FISH)

For IF, mouse brain tissues obtained 24 h after MCAO were fixed in 4% of paraformaldehyde for > 48 h and transferred to a 30% sucrose solution for dehydration. Ten-millimeter-thick paraffin coronal brain slices were acquired and incubated with a primary antibody against PPARg (Cat#bsm-33436 M; Bioss, Beijing, China) and UBE2V2 (Cat#10,689–1-AP; Proteintech, USA) overnight at 4 °C, followed by incubation with donkey anti-mouse Alexa Fluor-conjugated secondary antibodies (1:200, Jackson ImmunoResearch) for 1 h at room temperature. Finally, all slices were washed in PBS three times and counterstained with 4′,6-diamidino-2-phenyl in- dole dihydrochloride (DAPI; Cat#0100–20; SouthernBiotech, USA) for 5 min. Images were captured using a fluorescence microscope (Olympus, Japan), and the numbers of PPARg-positive and UBE2V2-positive cells in the infarction border cortex were counted and analyzed using ImageJ software.

For FISH, the Cy3-labeled miRNA-193a-5p probe was designed and synthesized by Servicebio. The FISH kit (Servicebio, Beijing, China) was used to detect probe signals in mouse brain tissues co-stained with anti-UBE2V2 and anti-Ly6G antibodies, according to the manufacturer’s instructions. Nuclei were stained with DAPI. All images were obtained with an LSM880 NLO (2 + 1 with BIG) confocal microscope system (Carl Zeiss).

### RNA Binding Protein Immunoprecipitation (RIP)

The RIP assay was conducted according to the protocol of the Magna RIP Kit (Millipore, MA, USA). Magnetic beads for RIP were incubated with primary antibodies against IgG and UBE2V2 in RIP wash buffer with rotation for 30 min at room temperature. The precoated magnetic beads were washed and then incubated with the cell lysates in RIP buffer overnight at 4 °C. The protein–RNA complexes were centrifuged, washed, and incubated with proteinase K in proteinase K buffer for 30 min at 55 °C to purify the protein-bound RNA. Finally, phenol–chloroform extraction and ethanol precipitation were performed according to the manufacturer’s protocol, and the abundance of protein-bound miR-193a-5p was determined by RT-qPCR.

### Immunoprecipitation and Western Blotting

Lysates of HL60 cells and brain tissue were prepared with IP lysis buffer (Cat#P0013, Beyotime, China), incubated for 2 h at 4 ℃, and centrifuged at 12,000 × *g* at 4 ℃ for 10 min. Protein G-Agarose (Cat#P3296, Sigma, USA) were prepared at 30 μl/tube for each sample (mixing thoroughly before using), and 500 μl IP Buffer (10 mM Tris–Cl, pH 7.5, 150 mM sodium chloride, 2 mM EDTA, 0.5% TritonX-100) was added to each sample, which was centrifuged at 2500 rpm at 4 ℃ for 5 min; the supernatant was discarded, and this was repeated three times. Then, 30 μl of prepared protein G-agarose was added to each lysate, with IP Buffer added to 500 μl, and samples were incubated at 4 °C for 3 h and centrifuged at 2500 rpm at 4 °C for 5 min, and the supernatant was retained. Next, 4.5 μl anti-PPARg (Cat#16,643–1-AP, Proteintech, USA) or IgG (Cat#2729S, Cell Signaling Technology, USA) of the same species was added to the supernatant of output or IgG groups, respectively; then, 30 μl prewashed protein G-agarose was added to each tube, which was incubated overnight at 4 °C. This was centrifuged at 2500 rpm for 5 min at 4 ℃, and the supernatant was discarded; then, 500 μl of IP Buffer was added to the precipitation, which was shaken gently. After centrifuging at 2500 rpm at 4 ℃ for 5 min, the supernatant was discarded, and this was repeated three times. SDS-PAGE 2 × loading buffer (30 μl) was added to the precipitate of each sample, which was homogenized. After heating at 90 ℃ for 5 min, the supernatant was centrifuged at 2500 rpm for 5 min and then subjected to western blotting.

Protein samples from input, IgG, and output groups were obtained to examine UBE2V2 expression levels by western blotting. Briefly, samples were subjected to electrophoresis and electrotransfer. The primary antibodies used were anti-UBE2V2 (Cat#10,689–1-AP, Proteintech, USA) and anti-PPARg (Cat#16,643–1-AP, Proteintech, USA). After incubation at 4 °C overnight, the blots were incubated with the corresponding horseradish peroxidase-conjugated secondary antibodies at room temperature for 1 h. Lastly, blots were visualized with an enhanced luminescence kit (Millipore, Billerica, MA, USA), and band intensity was analyzed using ImageJ software (National Institutes of Health, MD, USA).

### Statistical Analysis

Power analysis for sample sizes was performed based on treatment effect in preliminary data and previous studies. In order to achieve a power of test (1 − *β* = 0.9) and type I error rate (*α* = 0.05), the minimum sample size of each group in vivo and in vitro experiments was 5. All data were analyzed with SPSS 26 (SPSS Inc, Chicago, IL) and GraphPad Prism 8.4.0 (GraphPad Software, La Jolla, CA). Statistical significance was set at *p* < 0.05. Data distribution normality was assessed with the Shapiro–Wilk test (for sample size ≤ 50) and Kolmogorov–Smirnov test (for sample size > 50). Continuous variables were shown as means (SEM) for cases of normal distribution or medians (IQRs) otherwise. Except as stated, when Mann–Whitney *U* test was utilized in case of the abnormal data distribution, independent sample *t* test was utilized. One-way ANOVA with Tukey’s post hoc test was used for comparisons between multiple groups. Correlation analysis was performed using the Spearman correlation test. Receiver operator characteristic (ROC) curve analysis was applied to evaluate the predictive power (sensitivity and specificity) of miR-193a-5p levels for the diagnosis of ischemic stroke and the prognosis of patients receiving rtPA treatment.

## Results

### miR-193a-5p Is Decreased in Circulating Neutrophils of AIS Patients

**Fig. 1:**
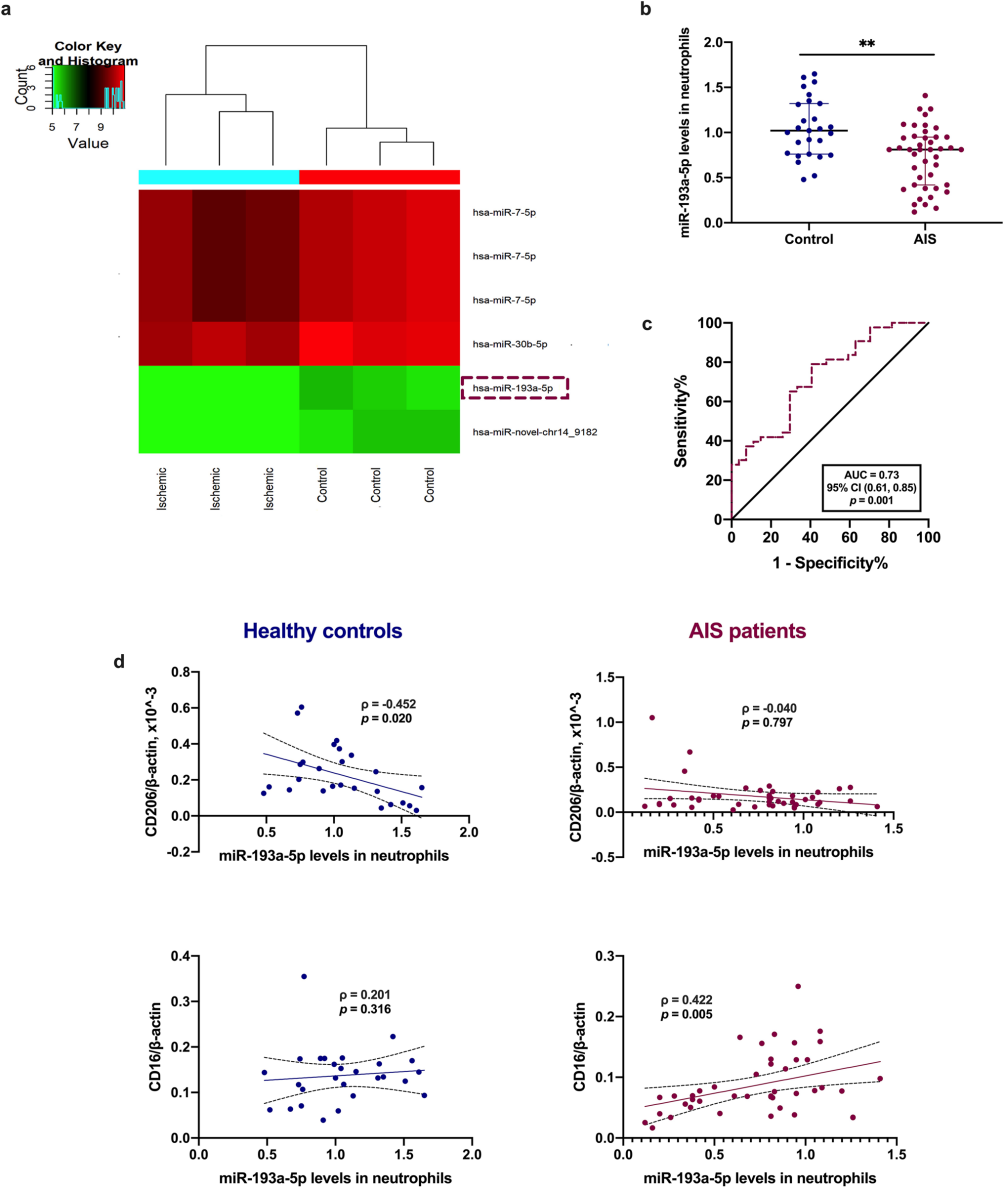
Expression of miR-193a-5p in circulating neutrophils of AIS patients. **a** Heatmap of the differentially expressed miRNA expression profile in ischemic stroke patients and controls using a miRNA sequencing; |fold-change|> 1.5, *p* < 0.05. **b** Validation of miR-193a-5p expression level in AIS patients (*n* = 43) and healthy controls (*n* = 27). **c** ROC analysis of miR-193a-5p in AIS patients and healthy controls. **d** Correlation analysis of the mRNA level of neutrophilic *CD16* or *CD206* and miR-193a-5p in neutrophils. AIS, acute ischemic stroke; AUC, area under ROC curve; CI, confidence interval; ROC, receiver operating characteristics. Bar charts reported in (**b**) show the median with interquartile range. ** *p* < 0.01 based on Man-Whitney *U* test (two-sided); the coefficient *ρ* (rho) was obtained via Spearman’s correlation analysis

To explore the expression and possible functions of miRNA in circulating neutrophils of AIS patients, we isolated neutrophils from the peripheral blood of AIS patients and healthy volunteers and used a microarray to detect differentially expressed miRNAs. Compared with expression in healthy controls, miR-193a-5p levels were significantly downregulated in peripheral neutrophils of AIS patients (Fig. 1a). RT-qPCR further verified that neutrophilic miR-193a-5p levels in AIS patients were significantly lower than those in healthy controls (Fig. 1b, *p* < 0.01). To determine its clinical relevance, an ROC curve was generated to assess the diagnostic value of neutrophilic miR-193a-5p in distinguishing AIS patients from healthy controls. As shown in Fig. 1c, the expression of neutrophilic miR-193a-5p had good diagnostic efficiency for AIS, with an area under the ROC curve of 0.73 ([0.61–0.85], *p* = 0.001).

### Higher Neutrophilic miR-193a-5p Is Associated with Better Outcomes for AIS Patients

**Fig. 2:**
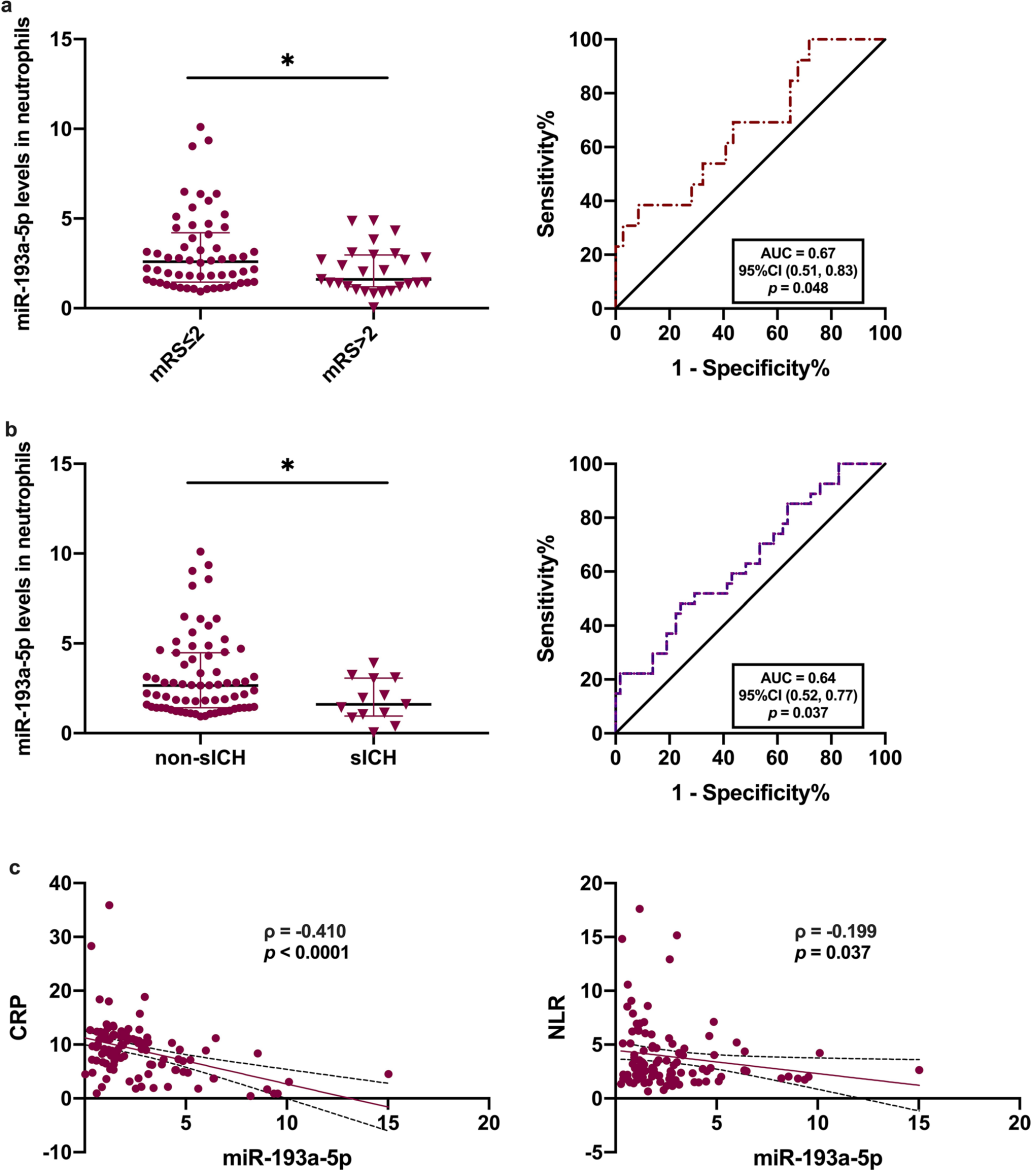
Association between neutrophilic miR-193a-5p and outcomes of AIS patients receiving rtPA treatment. **a** Neutrophilic miR-193a-5p according to an mRS ≤ 2 or mRS > 2 at 3 months. ROC analysis of neutrophilic miR-193a-5p and outcome at 3 months. **b** Neutrophilic miR-193a-5p according to the non-sICH or sICH group. ROC analysis of neutrophilic miR-193a-5p and sICH. **c** Correlation analysis of CRP and NLR with neutrophilic miR-193a-5p. *N* = 128. AIS, acute ischemic stroke; ROC, receiver operating characteristics; AUC, area under ROC curve; rtPA, recombinant tissue plasminogen activator; CI, confident interval; CRP, C-reactive protein; mRS, modified Rankin Scale; NLR, neutrophil-to-lymphocyte ratio; sICH, symptomatic intracerebral hemorrhage. Bar charts reported in (**a**–**b**) show the median with the interquartile range. *p < 0.05 based on the Man-Whitney *U* test (two-sided); the coefficient *ρ* (rho) was obtained via Spearman’s correlation analysis

To further examine the clinical significance of decreased miR-193a-5p levels in AIS patients at the superacute phase, we analyzed the association between neutrophilic miR-193a-5p levels and clinical outcomes including the mRS at 3 months and sICH in another subset of AIS patients receiving rtPA treatment. Compared to those in patients with an mRS > 2, neutrophilic miR-193a-5p levels were higher in the group with an mRS ≤ 2 and higher in non-sICH than in sICH patients (Fig. 2a–b, *p* < 0.05). ROC curves were created to assess the prognostic value of neutrophilic miR-193a-5p, with AUC values of 0.67 ([0.51, 0.83], *p* < 0.05) for distinguishing between unfavorable and favorable outcomes in AIS patients and 0.64 ([0.52, 0.77], *p* < 0.05) for distinguishing between sICH or non-sICH patients. Much clinical evidence suggests that higher circulating C-reactive protein (CRP) [26] and neutrophil–lymphocyte ratios (NLRs) [27] in IS are associated with an increased sICH risk and worse outcomes and are inflammatory indices for AIS patients. To determine the role of neutrophilic miR-193a-5p in neuroinflammation, we analyzed the correlation between its level and inflammation-associated parameters in AIS patients. Good correlations were found between the levels of CRP or NLR and neutrophilic miR-193a-5p (*ρ* =  − 0.410, *p* < 0.0001, *ρ* =  − 0.199, *p* < 0.05, respectively; Fig. 2c).

### AgomiR-193a-5p Protects the Brain from I/R Injury in Experimental Stroke

**Fig. 3:**
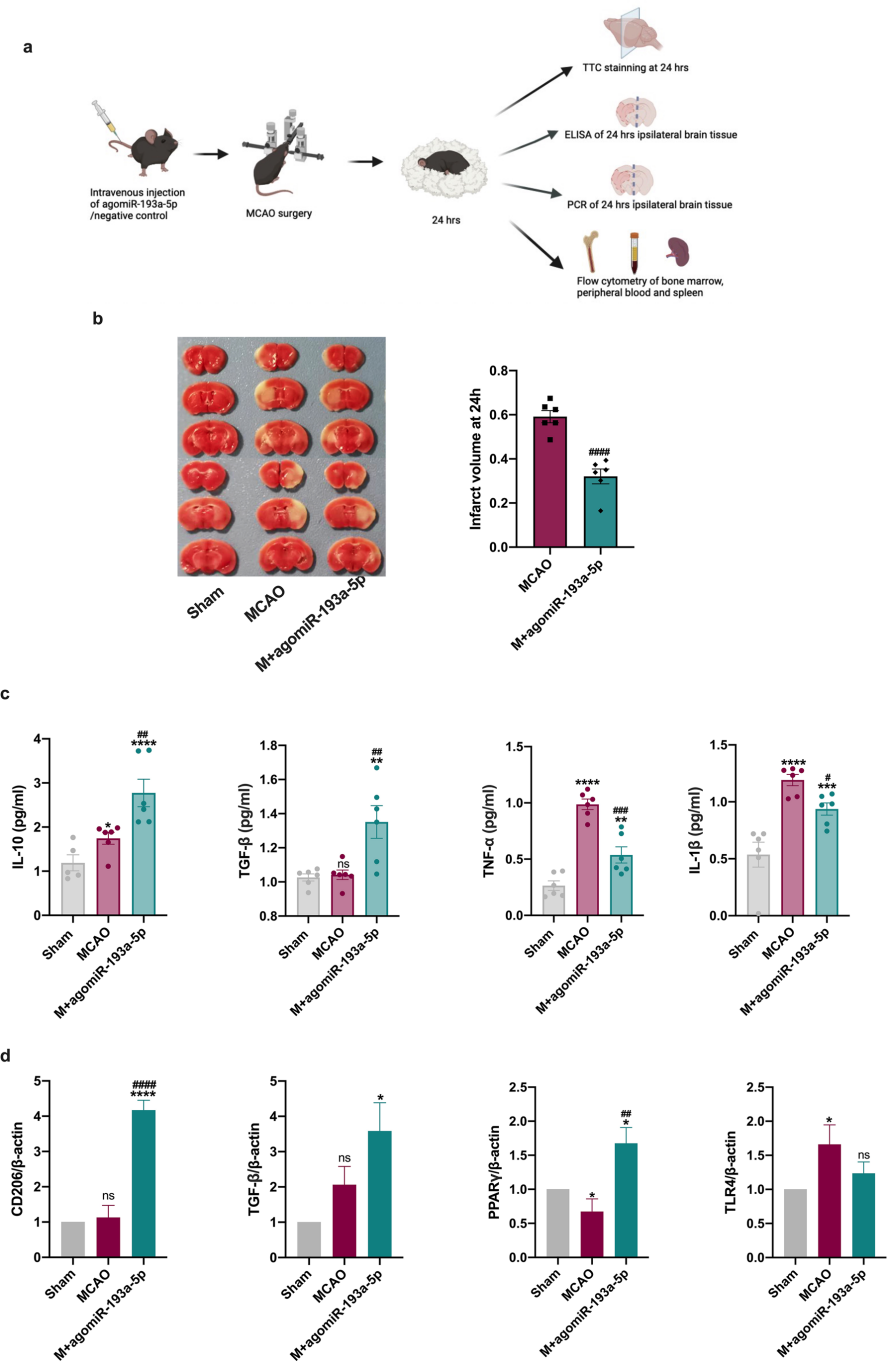
Effect of agomiR-193a-5p on brain injury in experimental stroke at the acute stage. **a** Brief illustration of the in vivo experiments at the acute stage. **b** Representative transverse slices from TTC staining of sham, MCAO, and MCAO plus agomiR-193a-5p groups at 24 h post-MCAO. Quantitative analysis of the infarct volume in the MCAO and MCAO + agomiR-193a-5p groups. **c** ELISA evaluation of IL-10, TGF-β, TNF-α, and IL-1β levels in the mouse ipsilateral brain tissue homogenate. **d** RT-qPCR analysis of *CD206*, *TGF-β*, *PPARγ*, and *TLR4* in the mouse ipsilateral brain tissue. Quantification of the relative value was based on the sham-operated control (set as 1). *N* = 6 per treatment arm. MCAO, transient middle cerebral artery occlusion; TTC, 2,3,5-triphenyltetrazolium chloride. Bar charts in (**c**–**d**) show the mean with SEM. *p < 0.05, **p < 0.01, ***p < 0.001, ****p < 0.0001, significantly different from sham group. ^#^*p* < 0.05, ^##^*p* < 0.01, ^###^*p* < 0.001, ^####^*p* < 0.0001, significantly different from MCAO group. ns, not significant

**Fig. 4:**
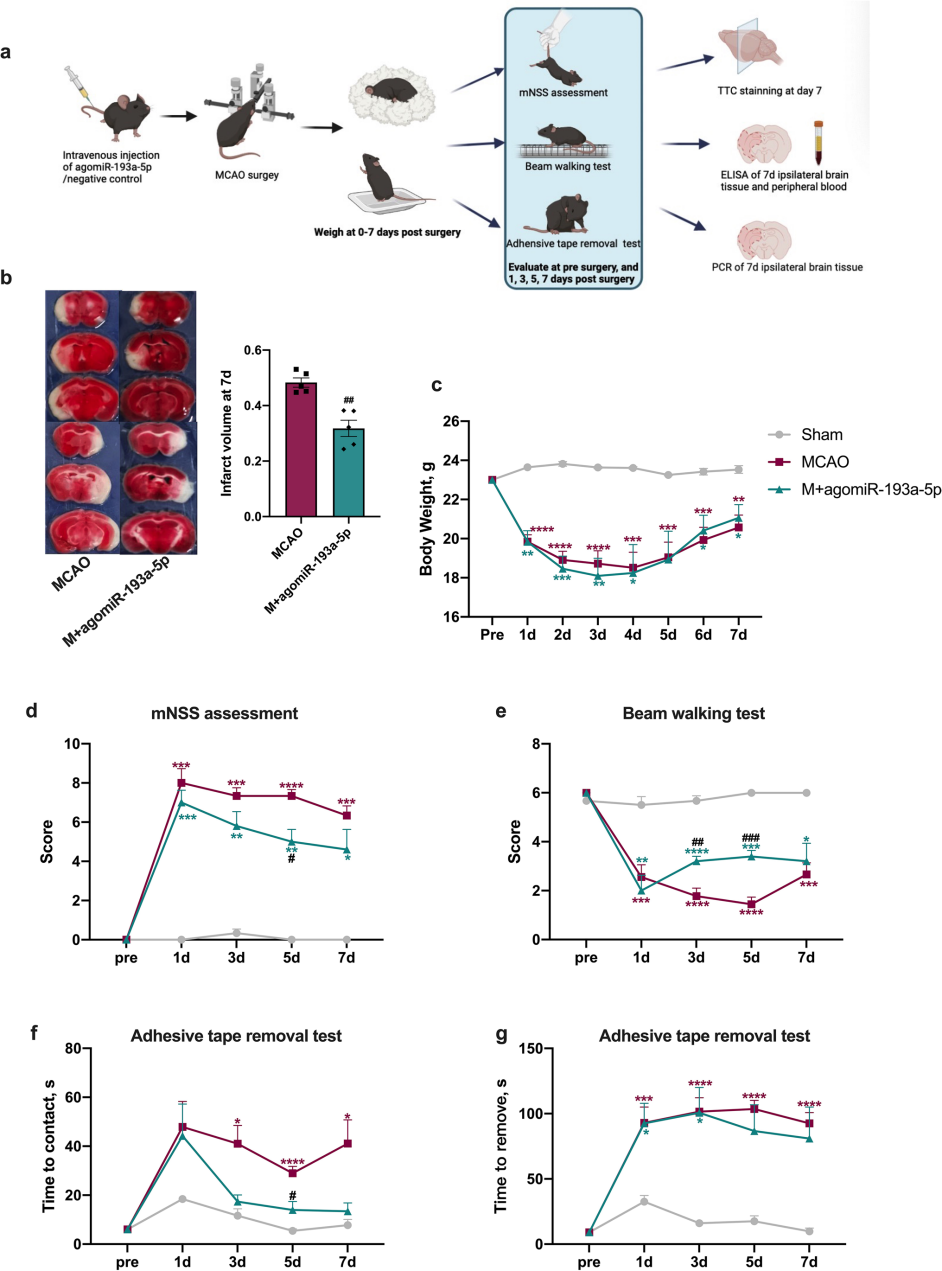
Effect of agomiR-193a-5p on functional recovery and brain injury in experimental stroke at the subacute stage. **a** Brief illustration of the in vivo experiments at the subacute stage. **b** Representative transverse slices from TTC staining of MCAO and MCAO + agomiR-193a-5p groups at 7 days post-MCAO. Quantitative analysis of the infarct volume in the MCAO and MCAO + agomiR-193a-5p groups. **c** Line chart of mouse body weights from pre-surgery to 1–7 days post-surgery. **d** Line chart of the mNSS, beam walking test score, time to contact/removal of the adhesive tape in the removal test for mice at pre-surgery, and 1, 3, 5, and 7 days post-surgery. *N* = 6 per treatment arm. MCAO, transient middle cerebral artery occlusion. TTC, 2,3,5-triphenyltetrazolium chloride; mNSS, mouse neurological severity score. Bar charts in (**b** − **g**) show the mean with the standard deviation. *p < 0.05, **p < 0.01, ***p < 0.001, ****p < 0.0001, significantly different from sham group. ^#^*p* < 0.05, ^##^*p* < 0.01, ^###^*p* < 0.001, ^####^*p* < 0.0001, significantly different from MCAO group. ns, not significant

To evaluate the role of miR-193a-5p in cerebral I/R injury, agomiR-193a-5p was delivered to the MCAO-model mice via tail vein injection before model induction, and the infarct volume at 24 h and 7 days post-I/R injury and neurological function scores at 1, 3, 5, and 7 days post-I/R injury (dpi) were examined (Figs. 3a, and 4a). TTC staining demonstrated that with the intravenous administration of agomiR-193a-5p, the infarct volume was smaller than that in the MCAO group at 24 h (*p* < 0.001; Fig. 3b) and 7 days (*p* < 0.01; Fig. 4b) post-I/R injury. As illustrated in Fig. 4c–g, agomiR-193a-5p injection resulted in a relatively lower mNSS at 5 dpi (*p* < 0.05, Fig. 4d) during repeated assessments, suggestive of good neurological behavior; the beam walking test indicated that miR-193a-5p-treated mice exhibited better neurological functional recovery after MCAO, as revealed by fewer instances of their hind legs falling off the beam during repeated tests at 3 and 5 dpi (*p* < 0.01, Fig. 2e). In comparison to MCAO-group mice, those treated with miR-193a-5p performed better in the adhesive tape removal test, as reflected by a decreased reaction latency (time until contacting but not removing the tape from the left forepaw at 5 dpi; *p* < 0.05, Fig. 2f, g). The difference in body weights between miR-193a-5p and MCAO groups was not statistically significant (Fig. 3c).

### Protective Role of AgomiR-193a-5p Is Correlated with Neutrophil N2 Transformation

**Fig. 5:**
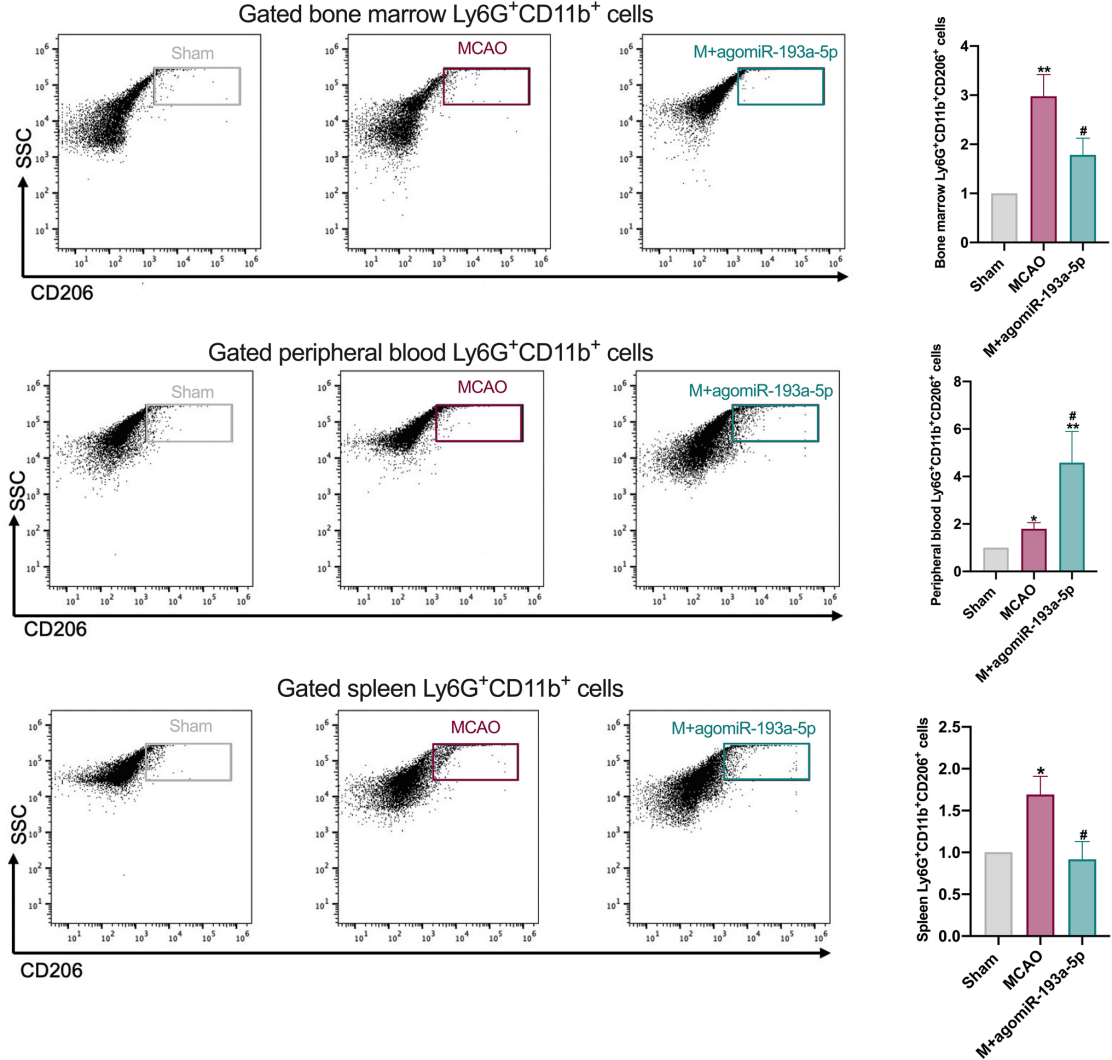
Flow cytometric analysis of the relative percentage of Ly6G^+^CD11b^+^CD206^+^ cells in the bone marrow, peripheral blood, and spleen at the acute stage of experimental stroke. *N* = 6 per treatment arm. Quantification of the relative percentage was based on the sham-operated control (set as 1). MCAO, transient middle cerebral artery occlusion. Bar charts show the mean with the SEM. *p < 0.05, **p < 0.01, significantly different from sham group. ^#^*p* < 0.05, significantly different from MCAO. ns, not significant

In determining whether miR-193a-5p could suppress I/R damage by modulating neutrophil phenotype switching, miR‐193a-5p levels were significantly associated with CD206 in the neutrophils of healthy controls (*ρ* =  − 0.452, *p* = 0.020) and CD16 in the neutrophils of AIS patients (*ρ* = 0.422, *p* = 0.005; Fig. 1d). To determine whether elevated miR‐193a-5p in blood could modulate neutrophilic N1–N2 shifts upon experimental stroke, we detected Ly6G^+^CD11b^+^CD206^+^ cells in the bone marrow, peripheral blood, and spleen 24 h after agomir-193a-5p intravenous injection by flow cytometry. Ly6G^+^CD11b^+^CD206^+^ cell proportions were increased in all three samples 24 h after I/R surgery, compared to those in the sham group; compared with those in the MCAO group, ratios of Ly6G^+^CD11b^+^CD206^+^ cells in the peripheral blood were further elevated, whereas ratios of Ly6G^+^CD11b^+^CD206^+^ cells in the bone marrow and spleen were decreased, with agomiR-193a-5p injection (Fig. 5). Meanwhile, we detected inflammatory cytokine levels in the ipsilateral brain tissue at 24 h by ELISA and PCR; TNF-α and IL-1β levels were upregulated in the MCAO group, which was reversed in the agomiR-193a-5p group, and IL-10 and TGF-β levels were also increased compared to those in the MCAO group (*p* < 0.05; Fig. 3c). Moreover, *CD206*, *TGFβ*, and *PPARγ* mRNA levels were also elevated in the ipsilateral brain tissue of the agomiR-193a-5p group compared to those in the MCAO group (*p* < 0.05; Fig. 3d). Additionally, we examined inflammatory cytokine levels in the ipsilateral brain tissue by ELISA and PCR and in the blood by ELISA at 7 dpi; TNF-α, IL-1β, IL-10, and TGF-β levels were all upregulated in the MCAO group, which was reversed in the agomiR-193a-5p group (*p* < 0.05; Fig. S1a). Only *CD16* mRNA levels were decreased in the brain tissue of the agomiR-193a-5p group compared to those in the MCAO group (*p* < 0.05; Fig. S1b). Peripheral blood IL-1β and IL-10 levels were lower in the of agomiR-193a-5p group than in the MCAO group (*p* < 0.05; Fig. S1c).

**Fig. 6:**
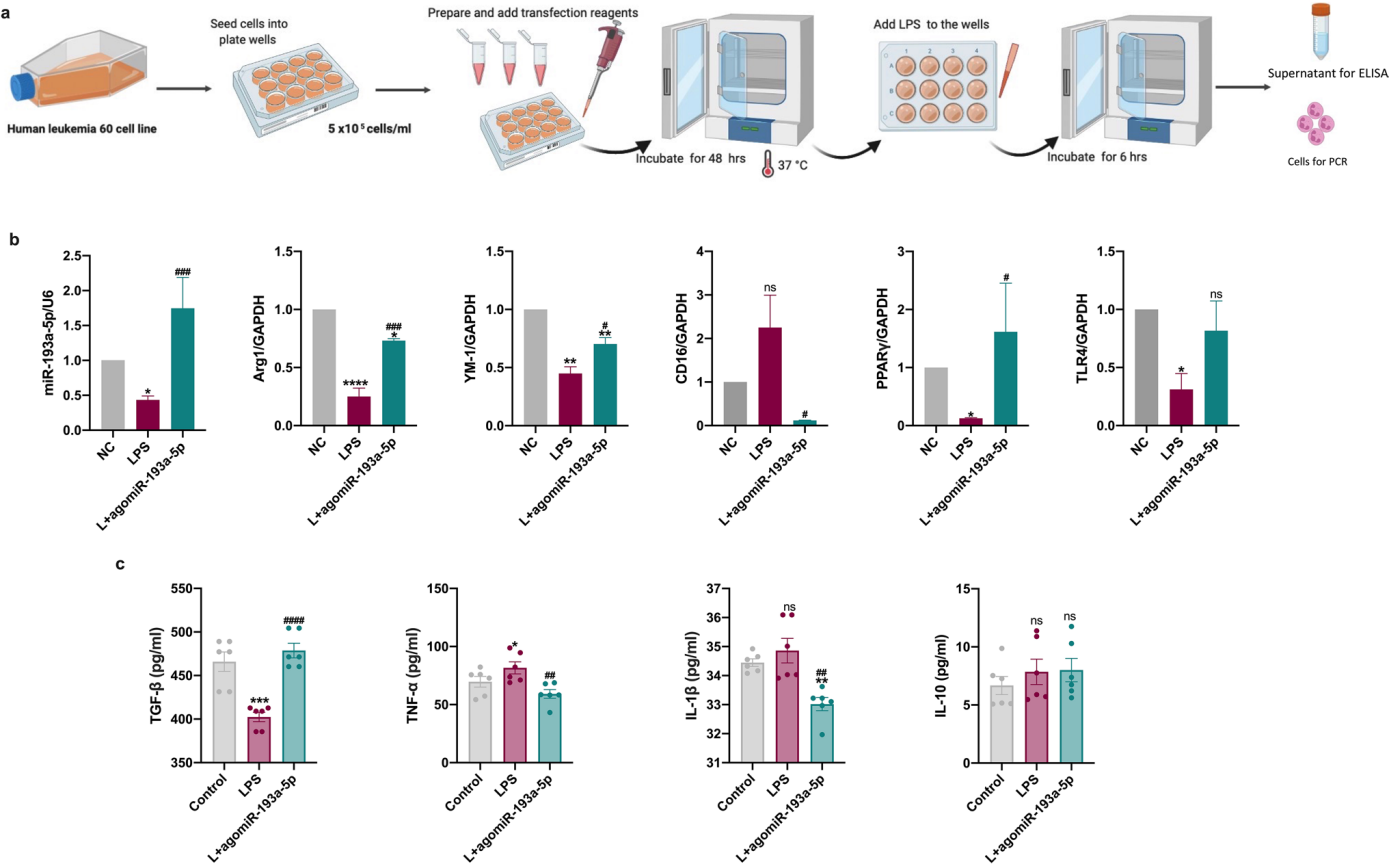
Effect of agomiR-193a-5p on the human neutrophil-like cell line with inflammation induced by LPS. **a** Brief illustration of the in vitro experiments. **b** RT-qPCR analysis of miR-193a-5p, *Arg1*, *YM1*, *CD16*, *PPARγ*, and *TLR4* in HL60 cells. **c** ELISA evaluation of TGF-β, TNF-α, IL-1β, and IL-10 levels in the supernatant of HL60 cells. Quantification of the relative value was based on the sham-operated control (set as 1). *N* = 6 per treatment arm. Bar charts reported in (**b**–**c**) show the mean with the SEM. *p < 0.05, **p < 0.01, ***p < 0.001, ****p < 0.0001, significantly different from control. ^#^*p* < 0.05, ^##^*p* < 0.01, ^###^*p* < 0.001, ^####^*p* < 0.0001, significantly different from LPS. ns, not significant

To further probe the possible effect of miR-193a-5p on neutrophils, we transfected HL60 human neutrophil-like cells with agomiR-193a-5p and then stimulated them with LPS to mimic inflammation after AIS (Fig. 6a). Compared to those in the LPS-only group, levels of arginase 1 (Arg1), chitinase 3-like 3 (Ym1), and PPARγ were increased, whereas CD16 levels were decreased in the miR-193a-5p overexpression group (*p* < 0.05; Fig. 6b). Levels of TNF-α and IL-1β were decreased, whereas those of TGF-β were elevated, in the supernatant of the agomiR-193a-5p-treated group, compared to those in the LPS-treated group (*p* < 0.05; Fig. 6c).

### miR-193a-5p Might Target Ubiquitin Conjugating Enzyme V2

**Fig. 7:**
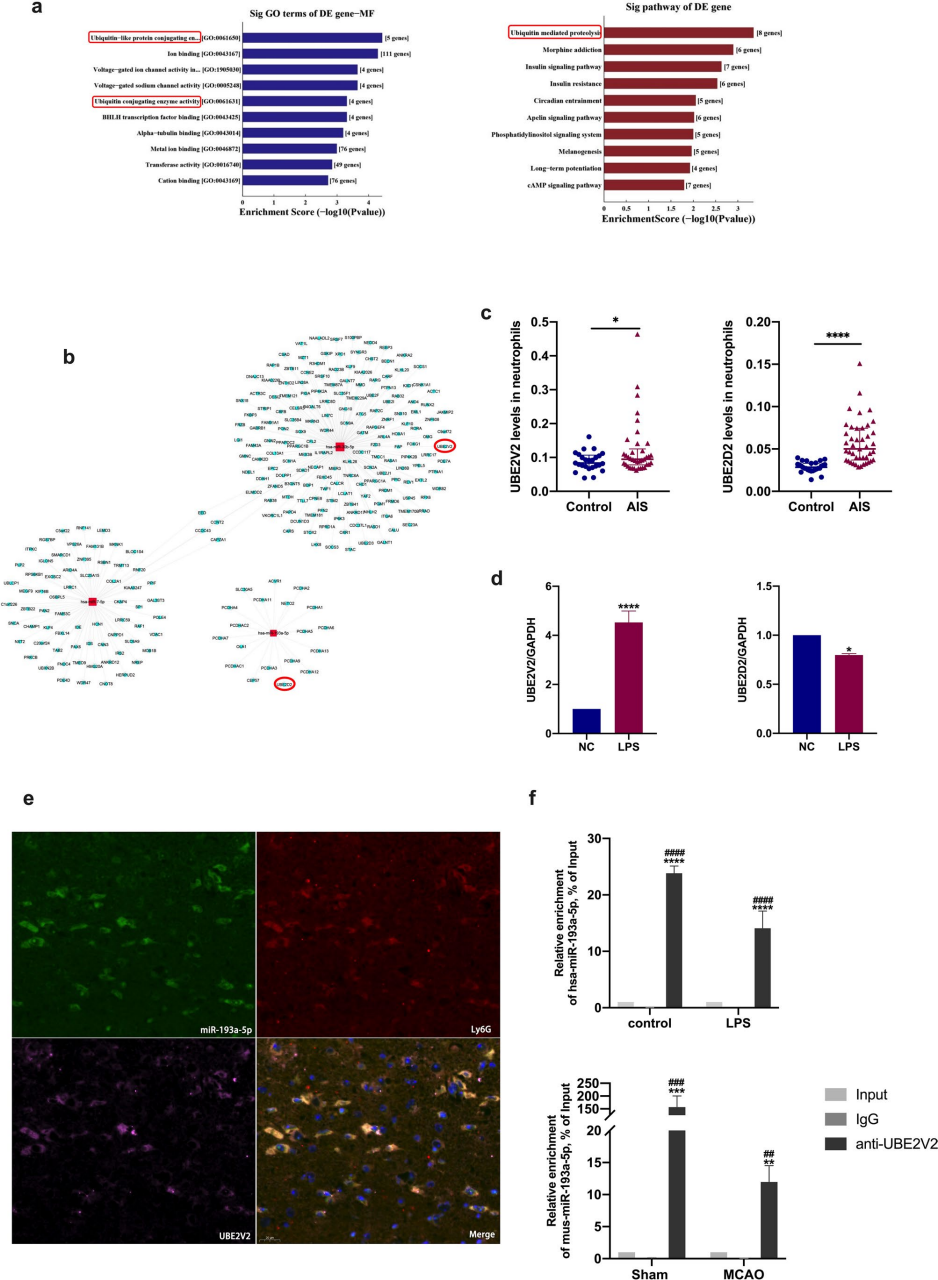
Prediction and validation of target genes of miR-193a-5p. **a** Biological functions (blue) and signaling (red) terms highlighted by GO and pathway analysis. **b** Prediction of putative target genes of miR-193a-5p. **c** Target gene validation by RT-qPCR in AIS patients (*n* = 43) and controls (*n* = 27). Analyzed by Man-Whitney *U* test (two-sided). **d** Target gene validation by RT-qPCR in HL60 cells (*n* = 6 per treatment arm). **e** Fluorescence in situ hybridization (FISH) analysis of miR-193a-5p (green), Ly6G (red), and UBE2V2 (purple) merged with DAPI (blue) present in C57BL/6 J mouse brain slices; bar = 20 μm. **f** RNA binding protein immunoprecipitation (RIP) of miR-193a-5p with UBE2V2 in HL60 cells and C57BL/6 J mouse brains (*n* = 4). AIS, acute ischemic stroke; MCAO, transient middle cerebral artery occlusion; NC, negative control. Bar charts reported in (**c**) show the median with the interquartile range; bar charts in (**d**, **f**) show the mean with the SEM. *p < 0.05, **p < 0.01, ***p < 0.001, ****p < 0.0001, significantly different from sham group. ^#^*p* < 0.05, ^##^*p* < 0.01, ^###^*p* < 0.001, ^####^*p* < 0.0001, significantly different from MCAO group

To further investigate the mechanism underlying the effect of miR-193a-5p on neutrophil phenotypic transformation with stroke, we performed bioinformatics analysis to predict the target genes of differentially expressed miRNAs. The top GO and KEGG terms included “ubiquitin-like protein conjugating enzyme” and “ubiquitin-mediated proteolysis” (Fig. 7a), and ubiquitin-conjugating enzyme V2 and D2 (UBE2D2 and UBE2V2) were identified by target-gene analysis (Fig. 7b). To verify bioinformatics analysis results, we first detected *UBE2V2* and *UBE2D2* mRNA levels in the circulating neutrophils of AIS patients and healthy controls; compared to those in the control groups, *UBE2V2* mRNA levels were upregulated in AIS patients and in LPS-stimulated HL-60 cells (Figs. 7c–d). We also performed FISH to observe the spatial distribution of miR-193a-5p and UBE2V2; as shown in Fig. 7e, miR-193a-5p and UBE2V2 were colocalized with Ly6G in both the cytoplasm and nucleus. miR-193a-5p was > 20-fold more abundant in the anti-UBE2V2 group than in the input group in the RIP experiment (*p* < 0.001, Fig. 7f).

### miR-193a-5p Might Induce Neutrophil N2 Transformation in Experimental Stroke via UBE2V2

**Fig. 8:**
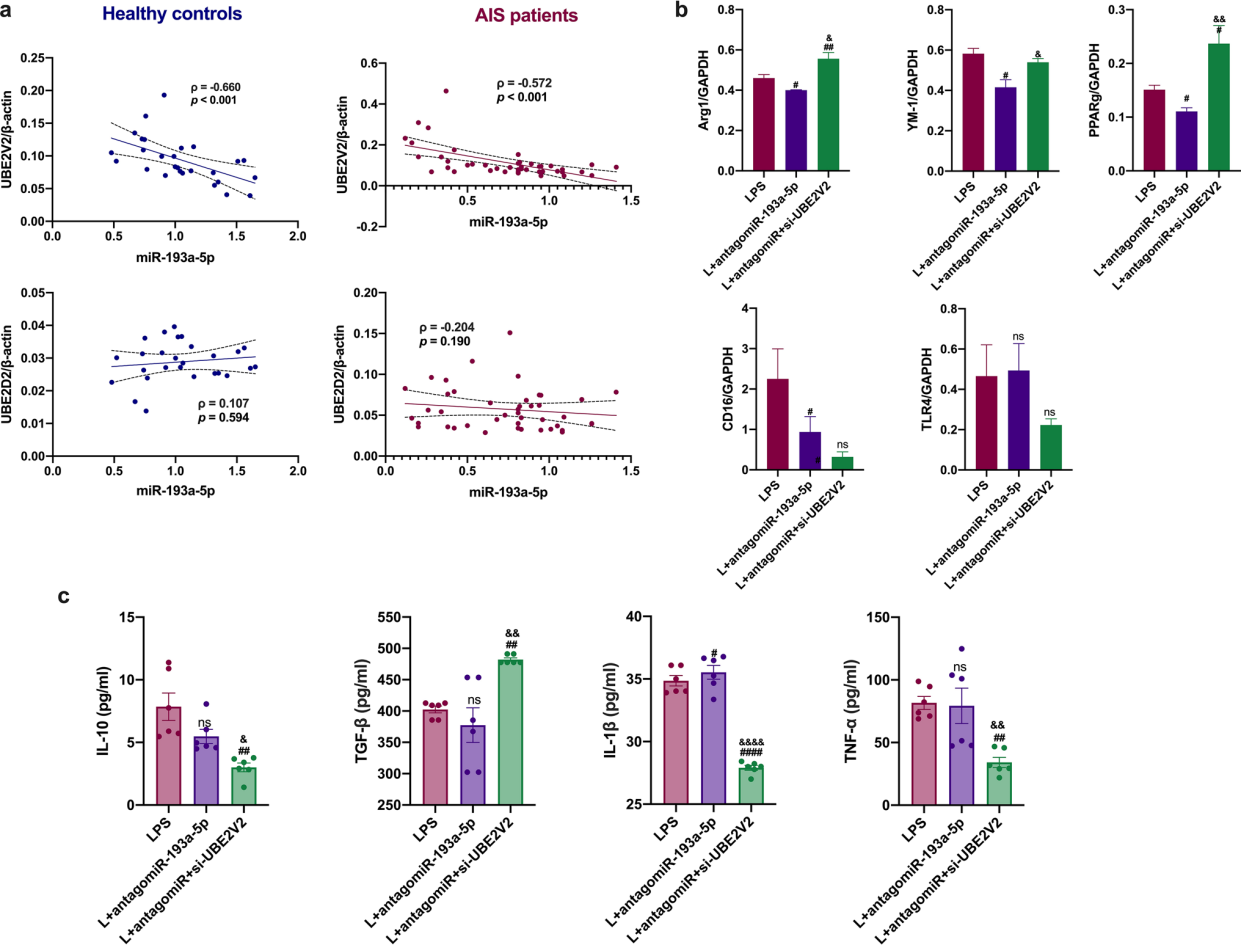
Expression of UBE2V2 in AIS patients and UBE2V2 silencing in human neutrophil-like cells. **a** Correlation analysis of the miRNA level of *UBE2V2* and *UBE2D2* with miR-193a-5p in neutrophils of AIS patients (*n* = 43) and healthy controls (*n* = 27). **b** RT-qPCR analysis of *Arg1*, *YM1*, *CD16*, *PPARγ*, and *TLR4* in HL60 cells; quantification of the relative value was based on the sham-operated control group (set as 1). **c** ELISA evaluation of TGF-β, TNF-α, IL-1β, and IL-10 in the supernatant of HL60 cells. *N* = 6 per treatment arm. The coefficient *ρ* (rho) was obtained via Spearman’s correlation analysis. AIS, acute ischemic stroke. Bar charts in (**b**–**c**) show the mean with the SEM. ^#^*p* < 0.05, ^##^*p* < 0.01, ^###^*p* < 0.001, ^####^*p* < 0.0001, significantly different from LPS group. ^&^*p* < 0.05, ^&&^*p* < 0.01, ^&&&^*p* < 0.001, ^&&&&^*p* < 0.0001, significantly different from LPS + antagomir-193a-5p group

**Fig. 9:**
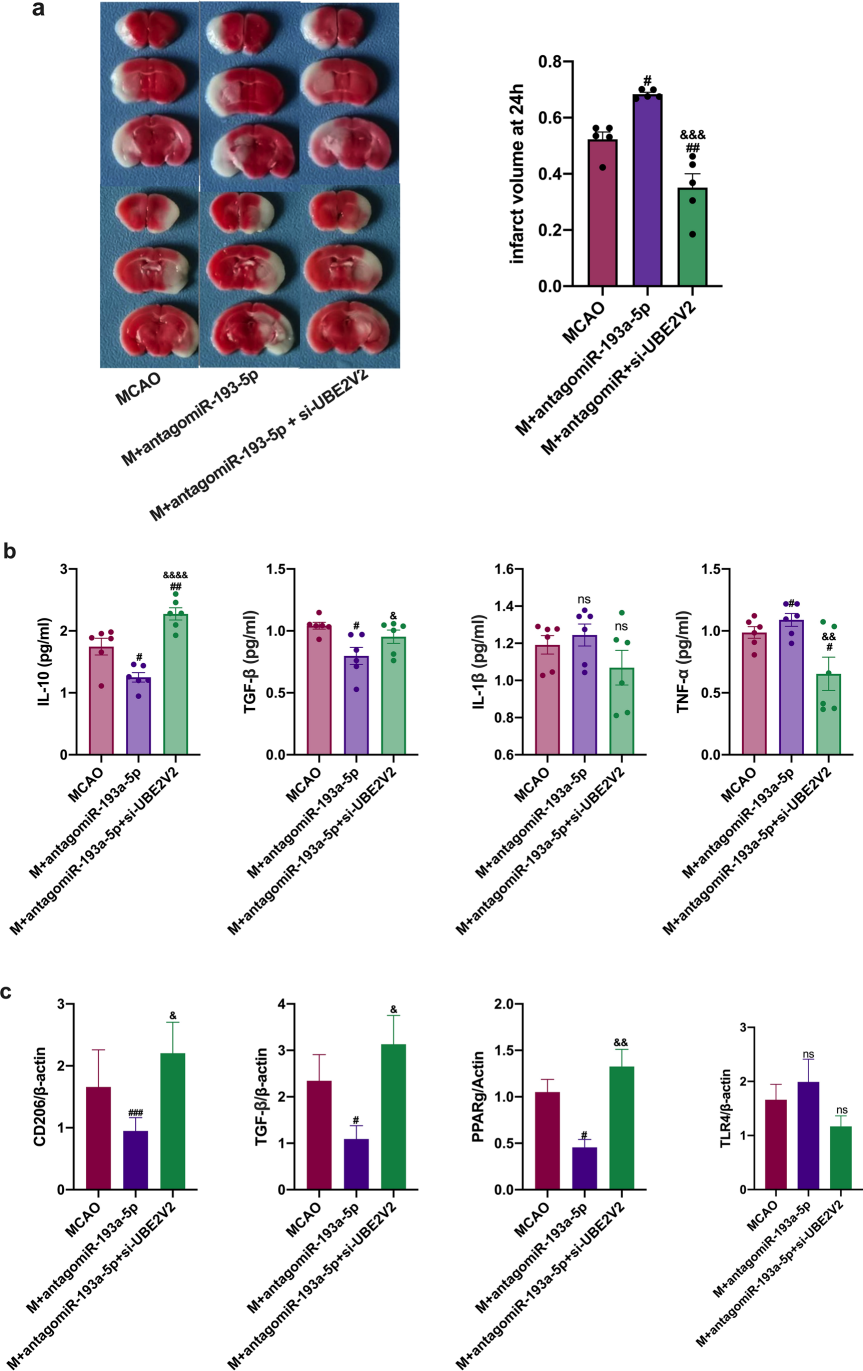
Effect of UBE2V2 silencing on experimental stroke. **a** Representative transverse slices from TTC staining of MCAO, MCAO + antagomiR-193a-5p, and MCAO + antagomiR-193a-5p + UBE2V2-siRNA groups at 24 h post-MCAO. Quantitative analysis of the infarct volume in the MCAO, MCAO + antagomiR-193a-5p, and MCAO + antagomiR-193a-5p + UBE2V2-siRNA groups. **b** ELISA evaluation of IL-10, TGF-β, TNF-α, and IL-1β levels in the mouse ipsilateral brain tissue homogenate. **d** RT-qPCR analysis of *CD206*, *TGF-β*, *PPARγ*, and *TLR4* in the mouse ipsilateral brain tissue. Quantification of relative values was based on the ham-operated control (set as 1). *N* = 6 per treatment arm. MCAO, transient middle cerebral artery occlusion. TTC, 2,3,5-triphenyltetrazolium chloride. Bar charts in (**a**–**c**) show the mean with the SEM. ^#^*p* < 0.05, ^##^*p* < 0.01, ^###^*p* < 0.001, ^####^*p* < 0.0001, significantly different from MCAO group. ^&^*p* < 0.05, ^&&^*p* < 0.01, ^&&&^*p* < 0.001, ^&&&&^*p* < 0.0001, significantly different from MCAO + antagomir-193a-5p group

miR-193a-5p levels were significantly correlated with *UBE2V2*, but not *UBE2D2*, mRNA levels in circulating neutrophils of AIS patients, and healthy controls (*ρ* =  − 0.660, *p* < 0.001; *ρ* =  − 0.572, *p* < 0.001, respectively; Fig. 8a). We hypothesized that miR-193a-5p might reprogram the neutrophil phenotype by acting on UBE2V2 to restore acute I/R damage and that UBE2V2 inhibition would reverse the harmful effect of antagomiR-193a-5p. To determine whether miR-193a-5p could affect neutrophilic N1–N2 shifts by targeting UBE2V2, we first detected the mRNA and protein levels of molecules representing N1/N2 phenotypes in vitro; in comparison to those in the LPS-treated group, *Arg1*, *Ym1*, and *PPARγ* mRNA levels were decreased in the antagomir-193a-5p-transfected group, and the additional administration of UBE2V2-siRNA reversed these effects (*p* < 0.05, Fig. 8b). TNF-α, IL-1β, and IL-10 levels were decreased, and TGF-β levels were elevated in the supernatant of the antagomiR-193a-5p plus UBE2V2 siRNA group compared to levels in the antagomiR-193a-5p group (*p* < 0.05, Fig. 8c). To confirm that miR-193a-5p might act on UBE2V2 to rescue acute I/R damage, we conducted in vivo studies using MCAO mice based on the intravenous transfusion of antagomiR-193a-5p and UBE2V2 siRNA. The cerebral infarct volume was greater in the antagomiR-193a-5p group than in the MCAO group, and antagomiR-193a-5p plus UBE2V2 siRNA transfusion reduced the infarct volume relative to that with antagomiR-193a-5p application (*p* < 0.05, Fig. 9a). IL-10 and TGF-β levels were increased in mice subjected to antagomir-193a-5p treatment compared to those in MCAO-model mice, which was reversed in mice co-administered antagomir-193a-5p and UBE2V2 (*p* < 0.05, Fig. 9b). *CD206*, *TGF-β*, and *PPARγ* mRNA levels were lower in the antagomiR-193a-5p group than in the MCAO group, which was reversed in the group co-administered antagomir-193a-5p and UBE2V2 (*p* < 0.05, Fig. 9c).

## Discussion

Reprogramming the neutrophil phenotype is expected to become an interventional target to reduce the inflammatory response and promote functional recovery after cerebral ischemic injury. In this study, we demonstrate that miR-193a-5p is decreased in circulating neutrophils of AIS patients and that higher miR-193a-5p levels are associated with better outcomes for AIS patients receiving rtPA treatment. At the molecular level, miR-193a-5p might regulate UBE2V2 to maintain the N1/N2 phenotypic balance and alleviate neuroinflammation-induced ischemic injury. These results suggest that miR-193a-5p might have a beneficial role in clinical AIS and is possibly associated with neuroinflammation. Functionally, we demonstrate that intravenous agomiR-193a-5p treatment has a protective effect on MCAO-model mice, as shown by the decreased infarct volume at 24 h and 7 days post-experimental I/R injury, as well as better neurological recovery. This could be linked to the skewed N2 circulating neutrophil subsets in the MCAO-model mice subjected to agomiR-193a-5p treatment; consistently, lower pro-inflammatory cytokine and higher anti-inflammatory cytokine levels were found in this group relative to those in the MCAO group. This was also initially suggested by the good correlation among neutrophilic CD16, CD206, and miR-193a-5p in patients and controls. We further show that agomiR-193a-5p is associated with better prognosis, as indicated by the improved performance in neurological function tests at 3 and 5 dpi and the smaller infarct volume at 7 dpi. Accordingly, pro-inflammatory and anti-inflammatory cytokine levels in the ipsilateral brain tissue were all lower with agomiR-193a-5p application, and circulating IL-1β levels were lower compared to those in the experimental I/R mice at 7 dpi, which could indicate that augmented neuroinflammation post-I/R injury is hampered in the agomiR-193a-5p-treated group at the subacute stage. Recent work suggests the involvement of miR-193a-5p in suppressing hepatocarcinogenesis [28] and human osteosarcoma cell metastasis [29], controlling cisplatin chemoresistance in primary bone tumors [30], reducing intestinal inflammation [31], and mediating protection against staphylococcal enterotoxin B-induced acute lung injury [32]. Nonetheless, the cell biological function of miR-193a-5p is largely unknown in ischemic injury.

For the in vivo study, agomiR-193a-5p or antagomiR-193a-5p was intravenously administered to mice. Thus, we cannot exclude the possible off-target effects in other organs, including infiltrating the brain through a compromised BBB post-I/R injury. Moreover, we could not exclude the possibility that the neuroprotective effect of miR-193a-5p is mediated through the targeting of neurons, glia, or other cell types from the bone marrow. Nonetheless, it is noteworthy that pre-surgery intravenous injection of agomiR-193a-5p or antagomiR-193a-5p first affected blood cells and that neutrophils represent the largest proportion and surge shortly after stroke injury, which make them the main target of transfection reagents/siRNA. In the brain tissue of mice subjected to agomiR-193a-5p treatment, *CD206*, *TGF-β*, and *PPARγ* mRNA levels were increased in comparison with those in MCAO mice, and miR-193a-5p was found to be co-localized with Ly6G in the MCAO ipsilateral brain tissue, together suggesting the involvement of miR-193a-5p in neutrophils infiltrating the cerebral parenchyma after experimental I/R injury. In previous studies, the protective effects of miR-193a-5p were shown based on a reduction in proliferation, survival, migration, and invasion [28], the uptake of bacterial products, and anti-inflammatory pathway targeting. Thus, it is possible that neutrophil phenotype switching is regulated by and functionally accounts for the effect of miR-193a-5p on I/R injury in certain conditions.

The question of the mechanism underlying the protective effect of miR-193a-5p remained. The key to probing the pathophysiological role of a microRNA is to identify its downstream gene(s). In this study, we examined the regulatory mechanism and downstream targets of miR-193a-5p via bioinformatic analysis. With this, we made the novel observation that miR-193a-5p is involved in pathways enriched in ubiquitin-conjugating enzyme activity. Ubiquitin conjugation (ubiquitination), a type of post-translational modification mediated by an enzymatic reaction cascade, precisely modulates protein functions in various cell types by affecting protein activity, stability, and interactomes [33]. The ubiquitin system was also reported to be responsible for fine-tuning the immune response [34]; as such, our results suggest that miR-193a-5p might target ubiquitin-conjugating enzyme activity during AIS for neutrophilic phenotype modulation. UBE2D2 and UBE2V2 were predicted to be two possible mRNA targets of miR-193a-5p in our bioinformatics analysis, and the upregulated expression of both was validated in neutrophils of AIS patients. UBE2V2, rather than UBE2D2, is highlighted as follows for its rather high abundance in neutrophils, and a previous study also reported that UBE2V2 is easily detected in immune cells [35]. Consistently, our results showed that *UBE2V2* mRNA levels were elevated in LPS-stimulated HL-60 cells relative to those in control samples, and UBE2V2 was spatially colocalized with miR-193a-5p in Ly6G^+^ cells. Furthermore, *UBE2V3*, but not *UBE2D2*, mRNA levels showed a good correlation with those of miR-193a-5p in neutrophils of AIS patients and controls. As such, UBE2V2 is likely a target of miR-193a-5p. This was further confirmed by FISH and RIP experiments. Interestingly, there is no known conventional 3′-UTR binding site between *UBE2V2* and miR-193a-5p (UGGGUCUUUGCGGGCGAGUGA). This is reminiscent of the unconventional role of miRNA-let-7 in activating TLR7 signaling in macrophages and microglia during neuroinflammation through a conserved GU-rich element in let-7 [36]. Accordingly, the FISH results showed that miR-193a-5p localizes to both the cytoplasm and nucleus, indicating that it might exert its anti-inflammatory effects via UBE2V2 in an unconventional style.

UBE2V2 (also known as MMS2) is a variant protein of the E2 ubiquitin conjugation enzyme; it forms an E2 complex with UBE2N (Ubc13) that then interacts with certain E3 ubiquitin ligases to catalyze poly-ubiquitination [37]. As the fate of a protein modified by ubiquitination is determined by the specific type of ubiquitin linkage, lysine 63 (K63)-linked ubiquitination was reported to regulate protein functions, such as activating proteins and promoting protein–protein interactions, and K48-linked ubiquitination generally targets substrate proteins for degradation via the proteasome [33, 38]. In immunoprecipitation and immunofluorescence studies (Fig. S2), we observed a possible interaction between UBE2V2 and PPARγ, and PPARγ is a recognized molecular switch for the neutrophil phenotype in IS [10]. Mechanistically, it is possible that this interaction is indicative of UBE2V2 interacting with an unknown E3 ligase to facilitate the K63- or K48-linked ubiquitination of PPARγ, functioning on PPARγ kinase and modulating pro- and anti-inflammatory cytokine profiles after cerebral ischemic injury. In line with this hypothesis, restoring miR-193a-5p increased PPARγ expression, whereas miR-193a-5p inhibition decreased PPARγ expression in LPS-treated HL60 cells and ipsilateral brain tissue at 24 h post-I/R injury. The combinational abrogation of miR-193a-5p and UBE2V2 reversed PPARγ expression relative to that in the miR-193a-5p inhibition group in vivo and in vitro. Moreover, mRNA levels of *Arg1* and *Ym1*, two markers of the N2 phenotype, increased and CD16, a marker of the N1 phenotype, decreased in the agomiR-193a-5p-administered group relative to levels in the LPS-stimulated HL60 group; meanwhile *Arg1* and *Ym1* mRNA levels decreased, and CD16 levels increased with antagomiR-193a-5p application, which was reversed by the additional knockdown of UBE2V2. In parallel, we found that the additional abrogation of UBE2V2 upregulated expression of the anti-inflammatory cytokines, IL-10 and TGF-β, but downregulated TNF-α and IL-1β expression in the supernatant of HL60 cells in comparison with levels in the antagomiR-193a-5p group.

Multiple studies have confirmed the role of miR-193a-5p in hampering inflammation [31, 32]. The present study provides a clue that miR-193a-5p exerts anti-inflammatory effects post-I/R injury. This is supported by the fact that agomiR-193a-5p intravenous injection upregulated anti-inflammatory cytokine and downregulated pro-inflammatory cytokine levels in brain tissues 24 h post-experimental I/R injury and also by the fact that intravenous agomiR-193a-5p transfusion suppressed all inflammation-associated cytokines in brain tissue at 7 dpi, though the mRNA level of *CD16* was still lower, and level of IL-1β in the peripheral blood was lower in the agmiR-193a-5p group than in the MCAO group at 7 dpi. Furthermore, the neuroprotective role of miR-193a-5p in the subacute stage was supported by the higher mNSS at 5 dpi, the better performance in the beam walking test at 3 and 5 dpi, and the better performance in the adhesive removal test at 5 dpi. Consistently, infarct volumes in the miR-193a-5p restoration group were smaller than those in the experimental I/R group at 24 h and 7 dpi. This evidence, based on the acute and subacute stages post-I/R injury, also supports the prognostic role of miR-193a-5p in AIS and its possibility of being translated to the clinic.

Accumulating evidence and careful examination stress the critical regulatory role of miRNAs in multiple aspects of IS pathogenesis, and modulation of neutrophil phenotypic transformation to augment restorative N2 subsets is a novel neurorestorative strategy to ameliorate the poor outcomes of stroke. Our findings suggest that the neuroprotective role of miR-193a-5p is likely established by restoring neutrophil N2 subsets, and this miR-193a-5p-induced effect is possibly linked to the UBE2V2-mediated-ubiquitination of PPARγ, which is much less studied than other signaling pathways targeting microRNAs. Unanswered questions arising from the current study include the following: (i) how miR-193a-5p affects UBE2V2, though the present findings suggest that miR-193a-5p affects UBE2V2 in an unconventional way; (2) which type of ubiquitination is mainly involved in the UBE2V2-mediated modification of PPARγ. Thus, there is a need to examine the ubiquitination status of PPARγ in the presence/absence of UBE2V2, with respect to its effect on neutrophil phenotypic transformation, in the future. Moreover, we must admit that there were limitations to this study. First, the agomiR-193a-5p was administered before MCAO model induction. This makes it difficult to translate its neuroprotective effect from basic research to clinical practice. Second, to better confirm the recovery using agomiR-193a-5p, we need to monitor the animals for a longer time after agomiR-193a-5p intervention, such as for 28 days. Based on the current findings with miR-193a-5p, our next plan is to postpone intervention to observe the neuroprotective effect of neutrophils and miR-193a-5p and to extend the observation period to 28 days.

Together, the present study demonstrates that agomiR-193a-5p has protective effects against experimental I/R injury, whereas miR-193a-5p inhibition exaggerates I/R injury. Moreover, both in vitro and in vivo data suggest that miR-193a-5p restores neutrophilic N2 phenotypes, which is associated with a reduction in I/R damage severity. Mechanistically, the effect of miR-193a-5p is likely induced, in part, through UBE2V2.

## Supplementary Information

Below is the link to the electronic supplementary material.

Supplementary file1 (JPG 3228 KB) Effect of agomiR-193a-5p on experimenta stroke at 7 d post-injury (dpi). a) ELISA evaluation of IL-10, TGF-β, TNF-α, and IL-1β levels in the mouse ipsilateral brain tissue homogenate at 7 d post-MCAO. b) RT-qPCR analysis of *CD206*, *TGF-β*, *Arg1*, and *CD16* in the mouse ipsilateral brain tissue. Quantification of relative values was based on the sham-operated control (set as 1). c) ELISA evaluation of IL-10, TGF-β, TNF-α, and IL-1β levels in the peripheral blood of mice at 7 d post-MCAO. homogenate. *N* = 6 per treatment arm. MCAO, transient middle cerebral artery occlusion. Bar charts in (a-c) show the mean with the SEM. * *p*  < 0.05, ** *p*  < 0.01, *** *p* < 0.001, **** *p* < 0.0001, significantly different from sham group. ^#^ *p*  < 0.05, ^##^ *p*  < 0.01, ^###^ *p* < 0.001, ^####^ *p* < 0.0001, significantly different from MCAO group. ns, not significant

Supplementary file2 (JPG 5399 KB) Immunoprecipitation and Immunofluorescence of PPARγ and UBE2V2 in mice. a) Extracts of the mouse brain tissue were subjected to immunoprecipitation using an anti-PPARγ antibody followed by Western blotting using an antibody against UBE2V2. Lanes of PPARγ detected in the input, IgG, and anti-PPARγ group. b) Immunofluorescence of PPARγ (green), UBE2V2 (red) merged with DAPI (blue) present in C57BL/6J mouse brain slice, bar = 20 μm.

Supplementary file3 (XLSX 56 KB)

Supplementary file4 (XLSX 68 KB)

## Data Availability

The data that support the current study are available from the corresponding author on reasonable request.
